# Gender disparity versus equality in acute stroke: a Middle Eastern country hospital-based study

**DOI:** 10.1186/s41983-023-00672-0

**Published:** 2023-06-01

**Authors:** John George, Hany Aref, Azza Abdel Nasser, Ayman Nasef, Ahmed Elbassiouny, Tamer Roushdy

**Affiliations:** 1 Neurology Specialist, Nasr City Insurance Hospital, Cairo, Egypt; 2 Neurology Department, Faculty of Medicine, Ain Shams University, 38 Abbasia, Cairo, PO 11591 Egypt

**Keywords:** Stroke, Gender difference, Female, Onset to door, Door to needle, Thrombolysis, Insurance hospital

## Abstract

**Background:**

Acute stroke management is well-established in developed countries with no gender difference. Yet, in developing countries there are reports on gender disparity in medical services including stroke services. Egypt, a developing low–middle-income country, heavily populated, in the Middle East is a good example to answer whether acute ischemic stroke service is provided equally to males and females or there is disparity in risk factors, onset to door (OTD), door to needle (DTN), and outcome. The current study was prospective observational analytical hospital-based study, on acute ischemic stroke cases admitted to Nasr city insurance hospital stroke unit between September 2020 and September 2022.

**Results:**

350 cases were included, 257 males and 93 females. Hypertension was the commonest risk factor 66% males and 81% females *P* = 0.011, atrial fibrillation was predominant in females *P* < 0.001, smoking was predominant in males *P* < 0.001. Median OTD in hours was 8.0 among both genders with minimum zero and maximum 96 h in males compared to minimum 1 and maximum 120 h in females, DTN was around 30 min with no significant difference. Median NIHSS on which rtPA was administered was 12.5 (6–13) in females compared to 10 (6–12) in males. Males who did not receive rtPA had a better mRS on discharge and on 90 days *P* = 0.01, 0.009, respectively, while there was no significant difference on discharge and 90 days between both genders on receiving rtPA.

**Conclusions:**

No gender disparity was found in DTN, discharge outcome, and 90 days among rtPA recipients. Females tended to have higher NIHSS and relatively delayed presentation to ER with less favorable outcome at discharge and 90 days in case of not receiving rtPA. Encouraging earlier arrival and conducting awareness campaigns for risk factors management is warranted.

## Background

In the past and prior to acute interventions and etiology-based diagnoses, stroke used to be managed as a single package disorder. Yet, with progress in diagnosis and investigational modalities, stroke no longer became a disorder of one-way management [1, 2].

Stroke etiology, diagnosis and management became greatly dependent on associated risks and whether it is controlled or not, onset of stroke and time of presentation to hospital—onset to door (OTD)—also has great impact on management [3].

In hospital time taken for confirmation of ischemic stroke and dealing with it—door to needle (DTN)—plays also an important role in outcome [4].

Meanwhile, sex and gender were not much given an important role in stroke, yet gender became an important factor in assessing risk of stroke and is embedded within a number of risk assessment scores as the CHADS2–VASc score [5].

Within the last decade stroke burden has changed a lot with a diversity between high-income countries (HIC) and low- (LIC) to low–middle-income countries (LMIC). Such stroke diversity is also evident among gender [6, 7].

Stroke management among males and females is reported to be equal in high-income countries and developed world. Yet, there is still few reported data about acute stroke risk factors, management and outcome within gender in LIC and LMIC in developing world. As a LMIC in the developing world within the middle east region with more than 102 million population, Egypt is a good example to answer the question of disparity versus equality in acute stroke risk factors, management, and outcome among males and females.

The aim behind this study was to assess acute ischemic stroke presentation among males and females presented to stroke unit of Nasr city insurance hospital in Cairo/Egypt as regards risk factors, severity of the presented stroke and its site, time of presentation to hospital (OTD), thrombolytic management including timing (DTN) and outcome so as to reach a conclusion whether there is disparity or equality in stroke presentation and management among males and females in a low–middle-income developing country.

## Methods

The current study is a prospective observational analytical hospital-based study, conducted on first ever or recurrent acute ischemic stroke patients, ≥ 18 years, of either gender, managed conservatively or administered thrombolysis, presented to the stroke unit of Nasr city insurance hospital—Cairo/Egypt along 2-year duration beginning from September 2020 to September 2022.

Nasr city insurance hospital is the largest among 39 health insurance hospitals, it is located in eastern Cairo and with its stroke unit and acute stroke management is considered a referral tertiary hospital in eastern Cairo.

After receiving an ethical approval from the responsible IRB, and obtaining a formal written informed consent from the patient or first order of kin, both genders were subjected to in-depth medical history including history of modifiable and non-modifiable vascular risk factors and neurological examination and assessment through the national institutes of health stroke scale (NIHSS) at baseline, and discharge.

A comparison was done between both gender’s OTD, DTN, site and size of infarction according to the supplying arterial territory, in hospital mortality, and outcome through the modified Rankin scale (mRS) with good outcome considered in case of mRS ≤ 2 and poor outcome > 2.

### Statistical analysis

All data were collected, tabulated and analyzed through the use of SPSS version 20. Unpaired student *T* test was used to compare between two groups in quantitative data, while paired student *T* test was used to compare between related sample. Pearson Chi-square was used for comparing categorical variables, median and IQR was used to compare non normality distributions. *P* value was considered significant if ≤ 0.05.

## Results

A total of 350 patients were admitted to the stroke unit along the study duration composed of 257 male patients accounting for (73.4%) and 93 female patients (26.6%). The mean age of males was 62.91 ± 11.28 versus 64.82 ± 13.22 in females. Hypertension was the commonest of all risk factors among both genders with more significance in females (*P* = 0.011). Diabetes Mellitus (DM) came second in males followed by dyslipidemia, while the reverse was true in case of females with dyslipidemia the second commonest risk factor followed by DM. Atrial fibrillation (AF) was statistically significant among females (*P* < 0.001), while smoking was the case among males (*P* < 0.001) (Table 1).

**Table 1:** Demographic distribution and risk factors of the studied cases

	Male		Female		*T* test	
	Mean	SD	Mean	SD	T	*P* value
Age	62.914 ± 11.287		64.828 ± 13.221		− 1.337	0.182

	*n*	Percentage (%)	*n*	Percentage (%)	Chi-square	
	257	73.43	93	26.57	*χ*^2^	*P* value
HTN	171	66.54	75	80.65	6.50	**0.011**
DM	125	48.64	40	43.01	0.868	0.35
AF	28	10.89	25	26.88	13.58	** < 0.001**
Dyslipidemia	111	43.19	42	45.16	0.108	0.74
ISH	76	29.57	20	21.51	2.23	0.13
Open heart surgery	8	3.11	2	2.15	0.22	0.63
Valve replacement	6	2.33	2	2.15	0.01	0.91
Current smoker	78	30.35	2	2.15	30.79	** < 0.001**
Ex-smoker	13	5.06	0	0	4.88	**0.02**
Stroke within 3 months	6	2.33	1	1.08	0.55	0.45
Stroke more than 3 months	47	18.29	13	13.98	0.89	0.34

The bold values indicate that they are statistically significant with *P* value < 0.05

HTN: hypertension, DM: diabetes Mellitus, AF: atrial fibrillation, ISH: ischemic heart

There was no significant difference in OTD among both genders with a median time of 8.0 h and a minimum of zero hours in males—stroke in hospital—and a maximum of 96 h compared to a minimum of 1 h in females and a maximum of 120 h.

DTN time was just exceeding 30 min among both genders with no significant difference being 35.78 min in males versus 37.5 in females (Table 2).

**Table 2:** Onset to door and door to needle among both genders

	Male	Female		
	Median (IQR)	Median (IQR)	Z	*P* value
OTD in hours	8.00 (7.5)	8.00 (7.85)	− 1.116	0.264

	Male	Female	*T* test	*P* value
	Mean ± SD	Mean ± SD		
DTN in minutes	35.784 ± 9.021	37.500 ± 7.272	− 0.654	0.515

OTD: onset to door, DTN: door to needle, IQR: interquartile range

Baseline NIHSS in males who received rtPA was 10 while who did not receive was 5 (*P* < 0.001) while baseline NIHSS in female patients who received rtPA was 12.5 compared to 6 for those who did not (*P* = 0.012).

Out of the entire 257 male patients, 189 presented with anterior circulation stroke and the majority were of middle cerebral artery territory infarction (57%) and 68 had posterior circulation stroke. As for female patients, 75 presented with anterior circulation stroke with the middle cerebral artery territory accounting for (67%) and 18 had posterior circulation stroke with insignificant difference between males and females in infarct site whether anterior or posterior as well as in infarct size either lacunar, territorial or total vessel occlusion distribution infarction. On comparing infarction presentation and going for rtPA again there was insignificant *P* value along the studied population (Table 3).

**Table 3:** Distribution of infarction according to site and size and thrombolysis decision

Imaging site and size		rtPA				Chi-square	
		Did not receive		Received			
		*n*	%	n	%	*χ*^2^	*P* value
**Male**							
MCA	Lacunar	41	19.90	11	21.57	11.568	0.072
MCA	Territorial	81	39.32	27	52.94		
MCA	Total	10	4.85	6	11.76		
ICA		2	0.97	0	0.00		
ACA		10	4.85	1	1.96		
Post-circulation	Lacunar	26	12.62	2	3.92		
Post-circulation	Territorial	36	17.48	4	7.84		
**Female**							
MCA	Lacunar	9	11.39	2	14.29	3.368	0.761
MCA	Territorial	38	48.10	8	57.14		
MCA	Total	10	12.66	3	21.43		
ICA		1	1.27	0	0.00		
ACA		4	5.06	0	0.00		
Post-circulation	Lacunar	7	8.86	0	0.00		
Post-circulation	Territorial	10	12.66	1	7.14		
Chi-square							
*χ*^2^		9.938		1.899			
*P* value		0.127		0.863			

MCA: middle cerebral artery, ICA: internal carotid artery, ACA: anterior cerebral artery, rtPA: recombinant tissue plasminogen activator

On discharge there was a significant difference in the NIHSS values among females compared to males who did not receive rtPA (*P* = 0.009), while there was insignificant difference among both genders on receiving rtPA on discharge or among same gender group on comparing treatment with rtPA to ordinary stroke care management (Table 4).

**Table 4:** National institutes of health stroke scale at baseline and discharge between both genders based on intervention

NIHSS baseline	rtPA		Mann–Whitney test	
	Did not receive	Received	Z	*P* value
	Median (IQR)	Median (IQR)		
Male	5 (4–9)	10 (6–12)	4.580	** < 0.001**
Female	6 (4–11)	12.5 (6–13)	2.499	**0.012**
Mann–Whitney test				
Z	2.157	1.585		
* P* value	**0.031**	0.113		
**NIHSS discharge**				
Male	3 (2–6)	3 (1–5)	0.889	0.374
Female	5 (3–10)	5 (0–7)	1.183	0.237
Mann–Whitney test				
Z	2.622	0.088		
*P* value	**0.009**	0.930		

The bold values indicate that they are statistically significant with *P* value < 0.05

NIHSS: national institutes of health stroke scale, rtPA: recombinant tissue plasminogen activator, IQR: interquartile range

In hospital stay for 7 days reached 75% of the entire sample with 72% males and 83% females. mRS 6 reached 9% in males with a total 23 out of 257 and 14% in females with a total 13 out of 93 with insignificant *P* value in both genders.

A total of 51% of male patients who received rtPA had good outcome at discharge (mRS 0–2) versus 49% who did not compared to 29% and 32% of female patients with insignificant difference among both genders and among each gender group. Meanwhile, there was statistically significant difference between percentage of male and female patients who did not receive rtPA and mRS at discharge (*P* = 0.010) (Table 5).

**Table 5:** Modified Rankin Scale on discharge among both genders

Sex	mRS on discharge	rtPA				Chi-Square	
		Did not receive		Received			
		n	%	n	%	*χ*^2^	*P* value
Male	Good outcome	100	48.54	26	50.98	0.097	0.755
	Poor outcome	106	51.46	25	49.02		
Female	Good outcome	25	31.65	4	28.57	0.052	0.819
	Poor outcome	54	68.35	10	71.43		
Chi-Square	*χ*^2^	6.622		2.220			
	*P* value	**0.010**		0.136			

The bold values indicate that they are statistically significant with *P* value < 0.05

mRS: modified Rankin Scale, Good outcome: mRS 0–2, Poor outcome: mRS 3–6, rtPA: recombinant tissue plasminogen activator

On 90-day follow-up, good outcome indicated by mRS ≤ 2 was reported in a total of 179 males (34 recipients of rtPA and 145 non-recipients) and a total of 50 females (8 recipients and 42 non-recipients) compared to a total of 55 males (8 recipients and 47 non) and a total of 30 females (1 recipients and 29 non) with poor outcome mRS > 2 with a significant difference between both genders who did not receive rtPA (Table 6).

**Table 6:** Modified Rankin Scale outcome at 3-month follow-up among both genders

Sex	Three-month mRS	rtPA				Chi-Square	
		Did not receive		Received			
		n	%	n	%	*χ*^2^	*P* value
Male	Good outcome	145	75.52	34	80.95	0.565	0.452
	Poor outcome	47	24.48	8	19.05		
Female	Good outcome	42	59.15	8	88.89	3.013	0.083
	Poor outcome	29	40.85	1	11.11		
Chi-Square	*χ*^2^	6.757		0.321			
	*P* value	**0.009**		0.571			

The bold values indicate that they are statistically significant with *P* value < 0.05

mRS: modified Rankin scale, Good outcome: mRS 0–2, Poor outcome: mRS 3–6, rtPA: recombinant tissue plasminogen activator

## Discussion

Stroke is one of the leading causes of death and disability worldwide. In the developed countries stroke has a consistent pathway and protocol of management that leads to unified outcome beside availability of awareness campaigns and EMS prenotifications. Such elements are not well-established in developing countries [8].

Besides, reports from developing countries points at disparity in medical care in general as well as in stroke care between males and females whether this is in medical care or medical expenditure [9, 10].

The current study was conducted along 2-years duration, and studied a total of 350 acute ischemic stroke cases, the majority of cases were males (73.4%). In a previous study, males accounted for the majority of cases (63.6%) admitted to Ain Shams university hospitals which is a major tertiary hospital in Cairo. Males also accounted for the majority of cases (65%) who sought medical care from acute ischemic stroke in another study conducted in a delta governorate in lower Egypt. Meanwhile, in Suhag governorate in upper Egypt male patients were 48.5% [11, 12].

Such variation in gender in seeking medical care in acute ischemic stroke was not the case in other studies. In an Austrian, and in a Canadian study, females were nearly equal to males [13, 14].

Variation in gender regarding seeking medical advice and admission could be attributed to financial aspects as males are usually the working power of the family and so they might seek medical attention so as to deal earlier with an illness to avoid any sick leave and absenteeism from work. Severity of illness which tended to be higher in females with a median NIHSS of 6 and 12.5 compared to 5 and 10 in females and males who did not receive and who received rtPA, respectively, highlights that females are admitted in case of severer strokes compared to males. Females’ symptoms sometimes are missed to be identified as a true stroke symptoms and are explained on basis of psychogenic ones that might dismiss female patients from ER and not admitting them.

Severity of stroke on baseline that is worse in females compared to males was discussed previously by Dehlendorff and colleagues who initially attributed it to age, where females are usually older in presentation of stroke than males but on adjustment of risk factors it was found that stroke in females has a tendency of being severe compared to males. Moreover, in our study age was statistically insignificant between males and females which agrees with Dehlendorff and colleagues that stroke in women tend in general to be more severe [15].

Along the current study hypertension was the commonest risk factor among both genders with significant *P* value towards females. Higher blood pressure measurements as well as diagnosis of hypertension in females compared to males could be attributed to more than one reason. Although it is speculated that androgens play a role in affecting natriuresis pressure as well as renin–angiotensin system which makes liability of hypertension more common in males than females yet, females have other risks along their life that expose them to hypertension. After puberty females are exposed to multiple and consecutive health situations that tend to increase their risk for developing high blood pressure and ultimately hypertension as pregnancy, hormonal therapies for birth control, and later on after menopause the protective role of estrogen in preventing hypertension is lost to the extent that during this age period females risk of developing hypertension exceeds that of males with about 52% of mortalities in females attributed to hypertension [16–18].Smoking whether a past habit or current smoking was more significant among males which could be considered as a habit that is generally more common in males. According to central agency for public mobilization and statistics issue released in 2021 the percentage of males who smokes in Egypt approached 36% compared to 0.3% females [19].

As for AF, it was more significant in females. AF in females could be attributed to undiagnosed or previous history of rheumatic heart disease which is reported to be more common in females than males [20].

Prenotification is not present in Egypt as reported in a previous study [21]. Absence of code stroke and prenotification did not affect OTD which also was insignificant among both genders despite females tended to arrive later than males although having a higher baseline NIHSS.

Although females tended to have a higher NIHSS than males at baseline yet, on receiving rtPA both improved to nearly an equal degree with insignificant *P* value (0.93). As for those who did not receive rtPA; NIHSS was statistically significant at baseline and was still significant on discharge with *P* = 0.03 and 0.009 among males and females, respectively.

Improvement in NIHSS on receiving rtPA highlights the beneficial role of such acute management therapy and confirms the importance of administering it to all eligible cases presenting in a time window of not more than 4.5 h. Despite the decline in chain of supplies due to the global economic crisis post COVID-19 that makes some cases with low NIHSS scores sometimes managed with loading doses of clopidogrel yet, this does not justify denying cases with low NIHSS from receiving rtPA [22].

Good outcome in cases treated with rtPA whether being males or females could be due to the success in administering rtPA in a proper time without delay with DTN reaching around 30 min in both females and males which highlights the concept of time is brain.

On assessing mRS at discharge and at 90-days follow-up, it was found that males who did not receive rtPA tended to have better outcome than females; this again could be attributed to more severe baseline NIHSS and a relative delayed presentation to ER in females that might be responsible for increasing the ischemic core and reducing the salvageable penumbra in females  compared to males rather than defective in-hospital management.

The current study has some limitations which include relative few numbers of cases despite conducting the study along 2-years interval from 2020 to 2022. This could be attributed to COVID-19 peaks which caused lockdown and curfew, avoidance behavior in some patients to seek help at hospitals for fear of catching infection, and transferring the hospital to quarantine one that extended few months within the study duration.

Also, the current study focused primarily on inpatient management with absence of post discharge follow-up visits except that on 90 days, so it is impossible to rule out the absence of an outpatient condensed physiotherapy courses role in poor functional outcome in female cases not treated with rtPA in comparison to males. We also admit that few cases performed neck ultrasound for assessment of carotid system and vertebral arteries as secondary to COVID-19 some assessments especially those with close proximity between physician and patient were excluded from routine practice.

Meanwhile, the current study strengths include that it is among few studies that compared gender in stroke in a developing country, being conducted in an insurance hospital rather than other studies conducted in stroke units of university hospitals proves that insurance hospitals with stroke units are as efficient as university hospitals.

## Conclusions

No gender disparity was found in DTN, discharge outcome, and 90 days among rtPA recipients. Females tended to have higher NIHSS and relatively delayed presentation to ER with less favorable outcome at discharge and 90 days in case of not receiving rtPA. Encouraging earlier arrival and conducting awareness campaigns for risk factors management is warranted.

## Data Availability

The data sets used and/or analyzed during the current study are available from the corresponding author on reasonable request.

## Abbreviations

OTD: Onset to door
DTN: Door to needle
HIC: High-income countries
LIC: Low-income countries
LMIC: Low–middle-income countries
AF: Atrial Fibrillation
HTN: Hypertension
DM: Diabetes mellitus
ISH: Ischemic heart disease
NIHSS: National institutes of health stroke scale
mRS: Modified Rankin scale
ER: Emergency room
EMS: Emergency medical services
IQR: Interquartile range
